# Simultaneous Quantification of Anticardiolipin IgG and IgM by Time Resolved Fluoroimmunoassay

**DOI:** 10.1371/journal.pone.0163682

**Published:** 2016-09-23

**Authors:** Zhigang Hu, Xiaoying Jing, Jie Liu, Mei Li, Yan Ye, Yu Chen

**Affiliations:** 1 Department of Laboratory Medicine, The First Affiliated Hospital, College of Medicine, Zhejiang University, Hangzhou, China; 2 Department of Laboratory Medicine, Affiliated Wuxi People’s Hospital of Nanjing Medical University, Wuxi, China; 3 Key Laboratory of Clinical In Vitro Diagnostic Techniques of Zhejiang Province, 79 Qingchun Road, Hangzhou 31003, China; Consiglio Nazionale delle Ricerche, ITALY

## Abstract

The autoimmune disease antiphospholipid syndrome (APS) is characterized by the presence of anticardiolipin antibodies (aCL), along with anti-β_2_-glycoprotein I (β_2_GPI) antibodies and lupus anticoagulant (LA). In this study, we developed a time-resolved fluoroimmunoassay (TRFIA) system for simultaneous quantification of aCL IgG and IgM. A 96-well microtiter plate precoated with the complex of cardiolipin from bovine heart and bovine β_2_GPI was incubated with the anticardiolipin IgG and IgM standard substance or serum, and the conjugate of Eu^3+^-labeled anti-human IgG and Sm^3+^-labeled anti-human IgM was pipetted to the wells to form a tipical double-antibody-sandwich immunoreactions; finally the fluorescent intensity of Eu^3+^ and Sm^3+^ was detected to reflect the quantity of anticardiolipin IgG and IgM. This assay showed a good relationship between fluorescence intensities and the concentration of anticardiolipin antibody(aCL) IgG and IgM, with a low-end sensitivity of 0.1 U/ml for IgG and 0.1 U/ml for IgM, respectively. The intra- and inter-assay coefficients of variation (CV) of the calibrators was 3.0% and 4.51% for IgG, and 2.76% and 4.45% for IgM. The average recovery was 100.38% for aCL IgG and 100.45% for aCL IgM. For serum samples, the results of our method showed a good correlation with those obtained with ELISA kit. Simultaneous detection of aCL-IgG and aCL-IgM in the same reaction well can optimize assay performance by avoiding potential influence of different reaction conditions-timing, and well-to-well difference in concentration and characteristics of cardiolipin antigen. The results of a combo aCL-IgG and aCL-IgM assay for the same sample are more consistent and more reliable. This dual-label time-resolved fluoroimmunoassay is sensitive for detecting aCL IgG and IgM across a wide concentration range with stable reagents and may assist in the clinical diagnosis of antiphospholipid syndrome.

## Introduction

Anticardiolipin antibodies (aCL) are autoimmune antibodies that target the negatively charged cardiolipin on the platelet and the cytomembrane of the endotheliocyte. They are the main antiphospholipid antibodies (aPL), along with anti-β_2_-glycoprotein I (β2GPI) antibodies and lupus anticoagulant (LA), that characterize the autoimmune disease antiphospholipid syndrome (APS). In 1983, Harris et al. developed the first aCL test [1]. In the same year, Graham Hughes and his team published their first report that showed an association of aCL with venous thrombosis, recurrent pregnancy loss, thrombocytopenia, and pulmonary hypertension, which are associated with APS [2,3]. Today, APS still remains a diagnostic challenge for clinicians, largely due to issues related to laboratory testing as well as the expanding range of reported clinical manifestations of APS. The revised 2006 laboratory criteria for APS [4] includes the presence of serum lupus anticoagulant, the presence of a medium or high titer of aCL IgG and/or IgM isotype, and the presence of anti-β2GPI IgG and/or IgM. Of the ‘criteria’ immunoassays for APS, aCL is the most sensitive while anti-β2GPI antibodies are considered highly specific with low sensitivity for APS [5]. Thus, in the initial diagnostic work-up of APS patients, a combination of an aCL ELISA and LA test can identify the majority of the patients [6–8]. However, employing active macromolecular enzyme as maker, the aCL ELISA itself has methodological and diagnostic limitations, particularly, its limited sensitivity, restricted linear range and poor measurement accuracy, which may make it fail to pick up the underlying patients and contribute to the explanation of ‘‘seronegative APS”(a case that patients do have clinical signs suggestive of APS but persistently test negative for antiphospholipid antibodies) [9].

TRFIA is a novel non-isotopic labeling technology developed in 1980s [10–12]. Having the fluorescent lanthanides (usually, Eu^3+^, Sm^3+^, Dy^3+^ and Tb^3+^) as tracers, TRFIA distinguishes itself by its wide detection range, high sensitivity (10^−18^ mol/ L), and less susceptibility to matrix interference, and has been well accepted as an ideal quantitative detection method for many analyses of clinical and biological importance. Moreover, the features that the detection windows and testing wavelengths of the four lanthanides were clearly distinguishable from each other makes it possible to be exploited in TRFIA to analyze even four analyses at a time. Of all the 15 lanthanides, Eu^3+^ is the most commonly used label in time-resolved fluorometry-based analysis because of its higher fluorescence yield and lower background than other lanthanide complexes. Sm^3+^ has been suggested as counterpart to Eu^3+^ in a dual-label system because it has equal excitation wavelengths (340nm) to Eu^3+^ as well as that it can be measured in the enhancement solution (β-NTA contained) optimized for Eu^3+^ detection [11]. Eu^3+^ and Tb^3+^ are another suggested pair in dual-label systems, but the enhancement solution optimized for Eu^3+^ detection cannot be used for Tb^3+^ since the later requires fluorinated aliphatic β-diketone for simultaneous detection [13], albeit that they form the most efficient fluorescent chelates [14]. With respect to the different isotypes of aCL, aCL-IgG and aCL-IgM are generally considered to be more strongly associated with the clinical manifestation of APS than aCL-IgA [15–17] and the detection of aCL-IgM antibodies theoretically reflects an early stage of the disease opposed to aCL-IgG which reflects a sustained class-switched immune response. To develop an assay system in which two or more analutes in one sample could be quantified simultaneously has the advantages of procedure simplification, increased throughput and reduced cost per test. However, none of the conventional methods, enzyme immunoassay (EIA) included, could completely meet the actual clinical demands. Considering the above, we developed an aCL IgG/IgM dual-label TRFIA for simultaneous detection of aCL IgG and IgM using the pair of lanthanides Eu^3+^ and Sm^3+^ as tracers in this study. Assay characteristics were determined and correlation and comparison to ELISA were performed. Clinical specificity was investigated with 510 samples from patients with other autoimmune manifistations. First, aCL antigen-the complex of cardiolipin from bovin heart and bovine β_2_GPI was precoated on the microtiter plate; second, the standard substance or serum was added into the wells of plate to incubate with aCL antigen; then, we pipetted conjugate of Eu^3+^-labeled anti-human IgG and Sm^3+^-labeled anti-human IgM to the wells to form a tipical double-antibody-sandwich immunoreactions; last, we added enhancement solution to each well and detected the fluorescent intensity using auto DELFIA 1235 TRFIA analyzer. Simultaneously detection for aCL-IgG and aCL-IgM in the same reaction well can avoid the influence brought out by different reaction conditions, different reaction duration and different concentration and characteristics of cardiolipin antigen in ELISA kit. So, the detection results of aCL-IgG and aCL-IgM for the same sample are more consistent and more reliable.

## Materials and Methods

### Chemicals and Reagents

Solid-phase antigen (the complex of cardiolipin from bovine heart plus bovine β_2_GPI), diethylenetriamine tetraacetic acid (DTTA), monoclonal rabbit anti-human IgM and IgG were purchased from Sigma (St. Louis, MO, USA). Cardiolipin solution used in this study was purchased from Sigma (St. Louis, MO, USA), which originated from bovin heart. 96-well polystyrene microtiter plates were obtained from Nunc International (Roskilde, Denmark). Bovine serum albumin (BSA) were supplied by the Institute of Biological Products Department of Health (Shanghai, China). Eu^3+^- and Sm^3+^- labeling kits were purchased from PE company (EG&G-Wallac, Finland). PD-10 column and Sepharose CL-6B column (1×40cm) were from the Pharmacia Company (NJ, USA). Enzyme-linked immunosorbent assay (ELISA) kits for aCL-IgG and aCL-IgM detection were purchased from Orgentec Diagnostika Gmbh (Naina, Germany). Auto DELFIA_1235_ TRFIA analyzer was supplied by Perkin-Elmer Life and Analytical Science/Wallac Oy (Turku, Finland). All additional chemicals and reagents used were of analytical grade.

Labeling buffer contained 50 mmol/L Na_2_CO_3_-NaHCO_3_ (pH 8.5) and 0.155 mol/L NaCl. Elution buffer included 50 mmol/L Tris-HC1 (pH 7.8), 0.9% NaC1, and 0.05% NaN_3._ Assay buffer consisted of 50 mmol/L Tris-HCl (pH 7.8), 0.9% NaC1, 0.2% BSA, 0.05% NaN_3_, 20 μmol/L DTPA, and 0.1% Tween-20. Washing solution was a Tris-HCl buffered saline solution (pH 7.8) which contained 0.9% NaCl, 0.2% Tween-20, and 0.05% NaN_3_. Enhancement solution was a 0.1 mol/L acetate-phthalate buffer (pH 3.2) that comprised of 0.1% triton X-100, 15 μmol /L β-naphthoyltriflu oroacetone (β-NTA), and 50 μmol/L tri-*n*-octylphosphine oxide.

### Solid-phase plate preparation

The complex of cardiolipin from bovine heart and bovine β_2_GPI was diluted to be coating buffer with 50 mmol/L Na_2_CO_3_-NaHCO_3_ buffer (pH 9.6), and 200μl coating buffer was added to each well of 96-well polystyrene microtiter plate and incubated overnight at 4°C. The coating buffer was discarded and each well was blocked with 200μl of 3g/L BSA for 2 hours on a miniorbital shaker. The blocking buffer was discarded and plates were dried under vacuum, stored at -20°C in a sealed plastic bag with desiccant until use.

### Labeling of anti-human IgM antibodies with Sm^3+^chelates and anti-human IgG antibodies with Eu^3+^chelates

Sm^3+^-labeling of monoclonal rabbit anti-human IgM was performed using a Sm^3+^-labeling kit strictly according to the manufacture’s instructions. Referring to the manual and previous report [18–19], the amount of each reagent and the reaction situations were followed in order to obtain the best binding efficiency of the labeled antibodies. Anti-human IgM (1ml of 8.1mg/ml) was loaded on a PD-10 column and eluted using sodium carbonate buffer (pH 8.5 50 mmol/L Na_2_CO_3_-NaHCO_3_) containing 0.155mol/L NaCl. Then the protein peak fractions were collected and concentrated to 2g/L. 500μl of the obtained purified second antibody was mixed with 0.2mg lyophilized Sm^3+^-DTTA, and stirred vigorously for 20 h at 25°C. The resulting mixture was then fractionated on a Sepharose CL-6B column (1×40cm) (Pharmacia, Piscataway, NJ, USA) and eluted with 80 mmol/L Tris-HCl buffer (pH 7.8). The absorbance values of the eluate were measured at 280 nm to obtain protein peak concentrations. After purification, added equal amount of AR glycerin before aliquoted and stored at -20°C until use.

Monoclonal rabbit anti-human IgG was labeled with Eu^3+^chelates using a Eu^3+^-labeling kit and the procedure was the same as the above-mentioned IgM labeling procedure.

### Clinical Serum Samples

52 serum specimens from patients with APS were collected for the correlation study. 50 serum samples from healthy volunteers referred to our hospital and 510 serum samples from patients with other auto-immune disease (scleroderma, nephrotic syndrome, ankylosing spondylitis, cerebral infarction, lupus nephritis, Sjogren syndrome, systemic lupus erythematosus and rheumatoid arthritis) were collected to test for clinical specificity. All serum specimens were separated at 2360g for 20min at room temperature after collection and stored at -70°C for further analysis. This specimen collection was approved by the Affiliated Wuxi People’s Hospital of Nanjing Medical University. All participants provided written informed consent to participate in this study.

### Assay principle and procedure of TRFIA for aCL-IgG and aCL-IgM

**Fig 1:**
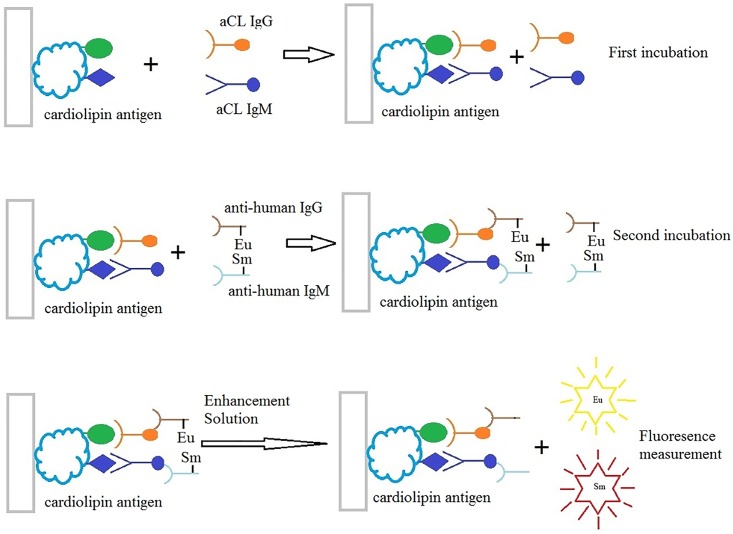
Schematic illustration of the dual-labeling TRFIA for simultaneous detection of aCL IgG and IgM. First, pipette 100μl/well pretreated standard substance or serum to microtiter plate that precoated with aCL antigen, and incubated the plate with shaking for 30 min at 25°C. A washing step removes unbound and unspecifically bound serum or plasma components. Second, added 100μl conjugate of buffer diluted Eu^3+^-labeled anti-human IgG and buffer diluted Sm^3+^-labeled anti-human IgM into each well, and incubated with shaking for 30min at 25°C. A second washing step removes unbound conjugate. Then, 200μl enhancement solution was pipette to each well and shaken for 5 min. Last, read the fluorescent intensity.

A two-site antibody-sandwich immune reaction was adopted to detect aCL IgM and IgG simultaneously in human serum with the dual-label TRFIA with the following steps. Specific antibodies (aCL IgM and IgG) in the patient sample bind to the antigen coated on the surface of the reaction wells. After incubation with shaking for 30 min at 25°C, a washing step removes unbound and non-specifically bound serum or plasma components. Eu^3+^-labeled anti-human IgG and Sm^3+^-labeled anti-human IgM conjugates are added into each well of the plate, incubated with shaking for 30min at 25°C. After incubation, a second washing step removes unbound conjugate. Next an enhancement solution is pipetted to each well and shaken for 5 min before reading the fluorescent intensity. Calibration curves were plotted and concentrations of aCL IgG and IgM in samples were determined using Multicalc software program. All procedures were automatically performed by auto DELFIA 1235 TRFIA analyzer with the process designed by our laboratory (Fig 1).

### Assay principle and procedure of ELISA for aCL-IgG and aCL-IgM

These ELISAs are indirect enzyme linked immune assay. Specific antibodies (aCL IgM or IgG) in the patient sample bind to the antigen (purified cardiolipin and beta-2-glycoprotien I) coated on the surface of the reaction wells. After incubation, a washing step removes unbound and non-specifically bound serum or plasma components. Next, the enzyme conjugate (HRP-labeled anti-human IgG or HRP-labeled anti-human IgM) is added and binds to the immobilized antibody-antigen-complexes. After incubation, a second washing step removes unbound enzyme conjugate. Then the substrate solution is added and the bound enzyme conjugate hydrolyses the substrate forming a blue coloured product. Addition of an acid stops the reaction generating a yellow end-product. The intensity of the yellow color correlates with the concentration of the antibody-antigen-complex and can be measured photometrically at 450 nm.

### Evaluation of the kits

#### Precision and linear range testing

Controls were made from three pools of mixed serum specimens with high, intermediate and low concentrations of aCL IgG and IgM were aliquoted and stored at -20°C. Intra- assay precision was determined with 25 reps in one assay testing all three controls; inter-assay precision was determined by running each conrol in duplicate for ten runs. The linear range of the assay was determined by testing serial dilutions of samples from patients with the highest aCL IgG and IgM concentration.

#### Sensitivity testing

The sensitivity of the assay was calculated from the mean fluorescent counts (*n* = 20) of the zero standard plus 2SD in the calibration curve.

#### Clinical applications

Plasma from 50 healthy volunteers was collected to verify the clinical specificity. Considering that the diagnosis of APS is complicated by the lack of a golden standard, results detected by the kits were correlated with the clinical criteria for APS.

#### Coefficient of recovery

Serial 2-fold dilution of three specimens with known concentrations were detected by the established dual-label TRFIA and the observed values were divided by the expected ones to calculated the coefficient of recovery.

#### Correlation test

Detected the aCL IgG and IgM concentrations of 52 serum specimens from patients with APS using the established dual-label TRFIA kit and the results were compared with those obtained with single-label ones as well as an ELISA kit.

#### Stability testing

Stability of the aCL IgG/IgM dual-label TRFIA kit and the commercial ELISA kit was determined by incubating the kit at 37°C for 7 days and then testing 20 aCL IgM and IgG positive sera respectively using the treated kits and kits that had been stored under normal storage conditions. Calculated the mean counts of fluorescent intensity and optical densities (ODs) respectively and then compared with values obtained with the one stored in routine conditions.

### Statistical analyses

Data were analyzed using the SPSS 13.0 software. Regression analysis and analysis of variance were used to calculate correlation and to test linearity, respectively. Inter-assay and intra-assay variation was calculated using the coefficient of variation.

## Results and Discussions

### Characterization of labeled antibodies

Eu^3+^-anti-human IgG antibody was purified by Sepharose CL-6B column and the first eluent peak was collected. According to the Eu^3+^ standard in the labeling kit as a reference, the first peak contained 77.4 μmol/L of Eu^3+^ and 9.5 μmol/L of protein, so each anti-human IgG may couple 8.1 Eu^3+^ ions on average. The labeling rate of Sm^3+^-anti-human IgM antibody was calculated in the same way. On average there may 7.9 Sm^3+^ ions per each anti-human IgM antibody according to its first peak obtained 77.4μmol/L of Sm^3+^ and 10.3 μmol/L of antibody.

### Calibration curve for quantification of aCL IgG and IgM

**Fig 2:**
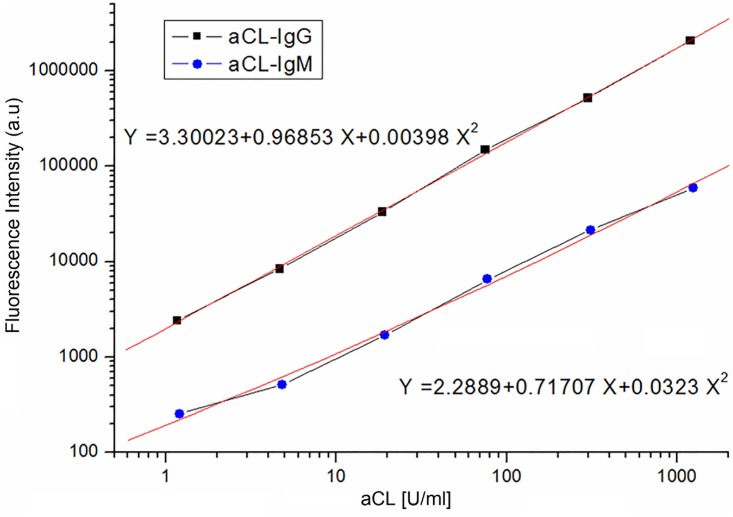
The calibration curves for aCL IgG and IgM using proposed dual-labeling TRFIA.

As showed in Fig 2, the time-resolved fluorescence intensities are directly proportional to the concentrations of aCL IgG and IgM. The line equation was Y = 3.30023+0.96853X+0.00398X^2^ for that of aCL IgG and Y = 2.2889+0.71707X+0.0323X^2^ for the calibration curve of aCL IgM, where X is the concentration of aCL IgG (U/ml) or aCL IgM (U/ml), and Y is fluorescence intensity.

### Assay Precision and detection limits

The precision of the assay was also analyzed by measuring three pools of mixed serum specimens with high, intermediate and low concentration of aCL IgM and IgG 25 times in one series (intra-assay) and in duplicate in ten different series. As shown in Table 1, the intra-assay CV ranged from 1.31% to 4.14% for aCL IgM and from 1.2% to 4.61% for aCL IgG. The inter-assay CV ranged from 2.29% to 6.97% for aCL IgM and from 2.26% to 7.34% for aCL IgG. The sensitivity of the assay, defined as the mean signal of the zero standard plus 2SD, was 0.1 U/ml for aCL IgM and 0.1 U/ml for aCL IgG.

**Table 1:** Intra- and Inter-assay precision of dual-label assay for aCL IgM and aCL IgG in serum of controls. It was analyzed by measuring three pools of mixed serum specimens with high, intermediate and low concentration of aCL IgM and IgG 25 times in one series (intra-assay) and in duplicate in ten different series.

	aCL IgM(U/ml)		aCL IgG(U/ml)	
	mean ± SD	CV (%)	mean ± SD	CV (%)
High				
Intra-batch(n = 25)	211.5±2.78	1.31	249.2±2.99	1.2
Inter-batch(n = 10)	210.9±4.83	2.29	249.2±5.64	2.26
Intermediate				
Intra-batch(n = 25)	71.53±2.02	2.82	82.77±2.63	3.18
Inter-batch(n = 10)	71.7±2.92	4.08	82.06±3.23	3.93
Low				
Intra-batch(n = 25)	14.46±0.60	4.14	15.62±0.72	4.61
Inter-batch(n = 10)	14.7±1.02	6.97	15.77±1.16	7.34

### Coefficient of recovery

Recovery with the dual-label TRFIA was determined using dilutions of three specimens with known concentrations of aCL IgG and IgM. The observed values were divided by the expected values to calculate the coefficient of recovery. As shown in Table 2, the average recovery rate of dual-label assay for aCL IgG was 100.38%. As shown in Table 3, the average recovery rate of dual-label assay for aCL IgM was 100.45%.

**Table 2:** Recovery rate of dual-label assay for aCL IgG.

Sample	Dilution	Observed(U/ml)	Expected(U/ml)	O/E (%)
1	1:100	167.5		
	1:200	84.65	83.75	101.07
	1:400	42.26	41.88	100.91
	1:800	20.23	20.94	96.61
2	1:100	73.6		
	1:200	35.24	36.8	95.76
	1:400	19.53	18.4	106.14
	1:800	8.75	9.2	95.11
3	1:100	94.7		
	1:200	46.17	47.35	97.51
	1:400	24.76	23.68	104.56
	1:800	12.53	11.84	105.83

**Table 3:** Recovery rate of dual-label assay for aCL IgM.

Sample	Dilution	Observed(U/ml)	Expected(U/ml)	O/E (%)
1	1:100	157.6		
	1:200	81.25	78.8	103.11
	1:400	37.84	39.4	96.04
	1:800	19.12	19.7	97.06
2	1:100	103.4		
	1:200	50.12	51.7	96.94
	1:400	27.54	25.85	106.54
	1:800	13.41	12.93	103.71
3	1:100	75.5		
	1:200	38.52	37.75	102.04
	1:400	18.15	18.88	96.13
	1:800	9.67	9.44	102.44

### Correlation with ELISA

**Fig 3:**
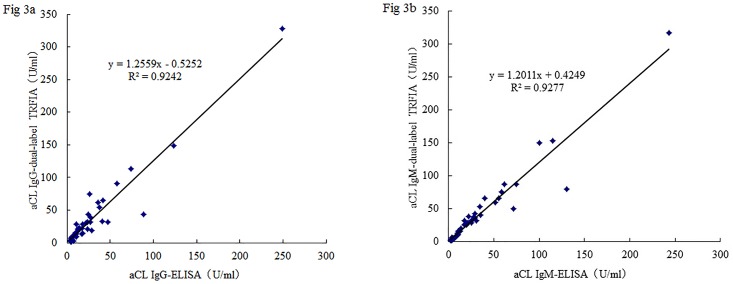
Correlation between ELISA and dual-label assay of aCL IgG (Fig 3a) and IgM (Fig 3b) in human sera (n = 52).

Concentration results of 52 serum samples determined by dual-label assay were compared with those obtained with ELISA kit. The scatterplots illustrated that the dual-label assay correlated well with ELISA (Fig 3a and 3b), and the correlation ratio between dual-label and ELISA was 0.924 for aCL IgG and 0.927 for aCL IgM.

### Comparison of linear range between ELISA and dual-label TRFIA

**Fig 4:**
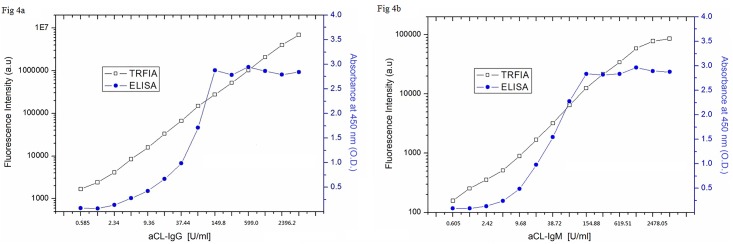
The comparison of liner range between ELISA and dual-label TRFIA for the detection of aCL IgG (Fig 4a) and IgM (Fig 4b), respectively.

Diluted a specimen with strong-positive aCL IgG from 4792.32 U/ml to 0.585 U/ml, and detected the serial dilutions with the established dual-label TRFIA as well as ELISA. The curve of detectable range of the two methods was showed in Fig 4a, from which we observed that for the established TRFIA kit there was a good liner range within 0.585 U/ml to 4792.32 U/ml for aCL IgG, whereas it was within 4.68 U/ml to 149.8 U/ml when using the commercial ELISA one. Then we diluted a specimen with strong-positive aCL IgM from 4956.1 U/ml to 0.605 U/ml and detected the serial dilutions. As showed in Fig 4b, we observed that the good liner range was lay within 4.84–154.88 U/ml when using a commercial ELISA kit, however, for the dual-label TRFIA one, it was lay within 0.605–4956.1 U/ml.

### Clinical application

The established aCL IgG/IgM dual-label TRFIA immunoassay was then applied to analyze the aCL IgG and IgM levels of serum specimens from 50 healthy volunteers and only one case of false-positive was produced, that is, the specificity of the assay was 98%. 510 serum specimens from patients with scleroderma, nephrotic syndrome, ankylosing spondylitis, cerebral infarction, lupus nephritis, Sjogren syndrome, systemic lupus erythematosus, rheumatoid arthritis were also collected and tested. Table 4 lists the positive rates of aCL IgG and aCL IgM.

**Table 4:** Clinical positive rate.

	Case number	ACA-IgG		ACA-IgM	
		Positive number	Positive rate(%)	Positive number	Positive rate(%)
scleroderma	24	4	16.67	1	4.17
Nephritic syndrome	36	1	2.78	4	11.11
Ankylosing spondylitis	14	1	7.14	2	14.29
Cerebral infarction	117	4	3.42	4	3.42
Lupus nephritis	33	5	15.15	9	27.27
Sicca syndrome	63	3	4.76	2	3.17
Systemic lupus erythematosus	153	12	7.84	13	8.5
Rheumatoid arthritis	70	6	8.57	7	10
total	510	34	6.67	42	8.24

### Stability of the TRFIA kits

The stability of the dual-label TRFIA kit and the commercial ELISA kits was investigated. After 7 days at 37°C, the stressed kit performance was compared to kits stored under normal conditions. There was a 15.8% decrease in the signal for aCL IgG and 14.7% decrease for aCL IgM. The ELISA kits demonstrated a greater decrease, 33.9% for aCL IgG and 37.6% for aCL IgM. In addition, we observed that the counts of fluorescent intensity obtained by the dual-label TRFIA kit was very stable even after overnight storage. The ODs obtained with the commercial ELISA one decreased quickly over time.

Additionally, because of its independence of the disintegration of the labeling compound, the stability of the aCL IgG/IgM dual-label TRFIA kits were substantially better than the ELISA ones.

### The strength and weakness of the dual-label TRFIA

As shown in Table 5, we have made a summarize of strength and weakness of the dual-label TRFIA method used for the anticardiolipin IgG and IgM detection.

**Table 5:** The strength and weakness of the dual-label TRFIA.

	Strength	Weakness
	Detection range is wide	Need special instruments
TRFIA	Detection objects are 2 kinds	
	Markers are stable and environmental protection	
	Signal results is stable in a long time	

## Conclusion

A new dual assay for anti-cardiolipin IgG and IgM has been developed utilizing a time resolved fluoroimmunoassay. The dual assay has good precision, a low analytical sensitivity, a good dilution recovery, a wide detection range and good correlation to an established method. Clinical specificity is 98% when evaluated against normal and greater than 90% when evaluated with samples from other disease states. The reagents are stable to heat stress and the fluorescence is stable over time. Compared to the commercial ELISA kits, the new assay had a larger assay range enabling better detection of low concentration samples as well as quantitation of high samples without further dilution and assay. Simultaneous detection of both aCL IgG and aCL IgM, as compared to two separate ELISAs, eliminates the possibility of differing reaction times and conditions, and possible sample instability. Time and labor are saved with running only one assay and not two. This new, dual TRFIA for anti-cardiolipin IgG and IgM has demonstrated good analytical and clinical performance and will enable the laboratory to provide analysis of these analytes in a routine manner.
